# *Tropheryma whipplei* endocarditis in Spain: Case reports of 17 prospective cases

**DOI:** 10.1097/MD.0000000000004058

**Published:** 2016-07-01

**Authors:** Lara García-Álvarez, María Mercedes Sanz, Mercedes Marín, MªCarmen Fariñas, Miguel Montejo, Josune Goikoetxea, Raquel Rodríguez García, Arístides de Alarcón, Manuel Almela, Núria Fernández-Hidalgo, María del Mar Alonso Socas, Miguel Ángel Goenaga, Enrique Navas, Luis Vicioso, José Antonio Oteo, the Spanish Collaboration on Endocarditis-Grupo de Apoyo al Manejo de la Endocarditis Infecciosa en España (GAMES)

**Affiliations:** a Departamento de Enfermedades Infecciosas, Hospital San Pedro-Centro de Investigación Biomédica de La Rioja (CIBIR); b Departamento de Enfermedades Infecciosas, Hospital San Pedro, Logroño; c Servicio de Microbiología Clínica y Enfermedades Infecciosas, Hospital General Universitario Gregorio Marañón, Instituto de Investigación Sanitaria Gregorio Marañón, Madrid; d Servicio de Enfermedades Infecciosas, Hospital Universitario Marqués de Valdecilla, Universidad de Cantabria, Santander; e Unidad de Enfermedades Infecciosas, Hospital Universitario de Cruces, Universidad del País Vasco; f Unidad de Enfermedades Infecciosas, Hospital Universitario de Cruces, Bilbao; g Servicio de Medicina Intensiva, Hospital Universitario Central de Asturias, Universidad de Oviedo, Oviedo; h Unidad de Gestión Clínica de Enfermedades Infecciosas, Microbiología y Medicina Preventiva, Hospital Universitario Virgen del Rocío, Sevilla; i Servicio de Microbiología y Parasitología, Hospital Clinic de Barcelona, Barcelona; j Servicio de Enfermedades Infecciosas, Hospital Universitario Vall d’Hebron, Universidad Autónoma de Barcelona, Barcelona; k Servicio de Enfermedades Infecciosas, Hospital Universitario de Canarias, Tenerife; l Servicio de Enfermedades Infecciosas, Hospital Universitario Donosti, San Sebastián; m Servicio de Enfermedades Infecciosas, Hospital Universitario Ramón y Cajal, Madrid; n Servicio de Anatomía Patológica, Hospital Clínico Universitario Virgen de la Victoria, Málaga, Spain.

**Keywords:** blood culture negative endocarditis, infectious endocarditis, *T. whipplei* endocarditis, *Tropheryma whipplei*

## Abstract

*Tropheryma whipplei* endocarditis is an uncommon condition with very few series and <90 cases reported in the literature. The aim of the study was to analyze the epidemiological, clinical, and outcome characteristics of 17 cases of *T. whipplei* endocarditis recruited in our country from a multicentric cohort from 25 Spanish hospitals from the Spanish Collaboration on Endocarditis—Grupo de Apoyo al Manejo de la Endocarditis infecciosa en España.

From a total of 3165 cases included in the cohort, 14.2% were diagnosed of blood culture negative endocarditis (BCNE) and 3.5% of these had *T. whipplei* endocarditis. This condition was more frequent in men. The average age was 60.3 years. Previous cardiac condition was present in 35.3% of the cases. The main clinical manifestation was cardiac failure (76.5%) while fever was only present in the 35.3%. Ecocardiography showed vegetations in 64.7% of patients. Surgery was performed in all but 1 cases and it allowed the diagnosis when molecular assays were performed. A broad range rRNA 16S polymerase chain reaction was used for first instance in all laboratories and different specific targets for *T. whipplei* were employed for confirmation. A concomitant Whipple disease was diagnosed in 11.9% of patients. All patients received specific antimicrobial treatment for at least 1 year, with no relapse and complete recovery.

*T. whipplei* endocarditis is an uncommon condition with an atypical presentation that must be considered in the diagnosis of BCNE. The prognosis is very good when an appropriate surgical management and antimicrobial-specific treatment is given.

## 1 Introduction

Blood culture negative endocarditis (BCNE) is a relative frequent condition among patients affected by infectious endocarditis (IE) representing 5% to 30% in great series.^[1–3]^ The main reasons for this condition are the previous administration of antimicrobials and fastidiously culture microorganisms. Anyway, in the past decades, the application of certain tools as automated blood cultures, molecular assays, immunohistochemistry (IHC), and serology has improved the diagnosis of this condition and has involved new agents.^[4]^ These facts have been incorporated in new guidelines.^[5,6]^

*Tropheryma whipplei*, formerly *Tropheryma whippelii*, is an intracellular gram-positive Actinobacteria ubiquitous in the environment that is involved in a large variety of clinical forms.^[7,8]^ First implication of *T. whipplei* as causative agent of infective endocarditis was reported from Switzerland in 1997, in a patient with BCNE using a broad-range polymerase chain reaction (PCR) followed by sequencing.^[9]^ First stable cultivation of the bacterium of Whipple disease (WD) was carried out in 2000, from the mitral valve of a patient with BCNE.^[7]^ The knowledge of the genome of *T. whipplei* has permitted the development of specific and sensible tools for diagnosis and have involved this microorganism in a broad spectrum of clinical conditions.^[10,11]^

Sporadic cases of *T. whipplei* endocarditis have been reported from different countries,^[12]^ but there are few published series of *T. whipplei* endocarditis.^[13,14]^ In this article, we describe the epidemiological, clinical, and outcome characteristics of 17 cases of *T. whipplei* endocarditis diagnosed in several hospitals from Spain. Some cases have been previously reported.^[15,16]^

## 2 Patients and methods

### 2.1 Patients’ recruitment

All but 1 patients diagnosed of *T. whipplei* endocarditis were recruited from a registry of the “Spanish Collaboration on Endocarditis—Grupo de Apoyo al Manejo de la Endocarditis infecciosa en España” (GAMES). In this registry, consecutive patients with IE were included between January 1, 2008 and December 31, 2014 in 25 Spanish hospitals. Multidisciplinary teams prospectively completed a standardized case report form containing epidemiological, clinical, biological (including main hematological and biochemical values), and therapeutical data for each patient. Regional and local ethics committees approved the study (Comité Ético para la Investigación Clínica-Regional de la Consejería de Sanidad de la Comunidad de Madrid, code: 18/07; Date: January 11, 2008) and patients gave their informed consent for entering the cohort.^[3]^

A routinary protocol that includes serology to *Coxiella burnetii*, *Bartonella* spp., *Legionella* spp., and *Brucella* spp. was completed in all patients with BCNE. When surgery was performed, all hospitals had the opportunity to send the valves to the referral centers located at Madrid, Catalonia and La Rioja for molecular studies.

### 2.2 Definitions

IE was defined according to the modified Duke criteria.^[17]^ Definitive *T. whipplei* endocarditis was considered if positive results of periodic acid-Schiff (PAS) staining and/or specific IHC test using specific antibodies against *T. whipplei* and/or 2 positive results of PCR assays targeting 2 different sequences in a cardiac valve specimen were met.^[18]^

## 3 Results

### 3.1 Epidemiological data

A total of 3165 cases of IE were recorded in the GAMES Cohort between 2008 and 2014. From the total, 451 (14.2%) were diagnosed of BCNE and 16 (3.5%) of these had IE by *T. whipplei*. One case was added from a hospital not included in the GAMES group. Main epidemiological, clinical, and outcome characteristics are shown in Table 1.

**Table 1:**
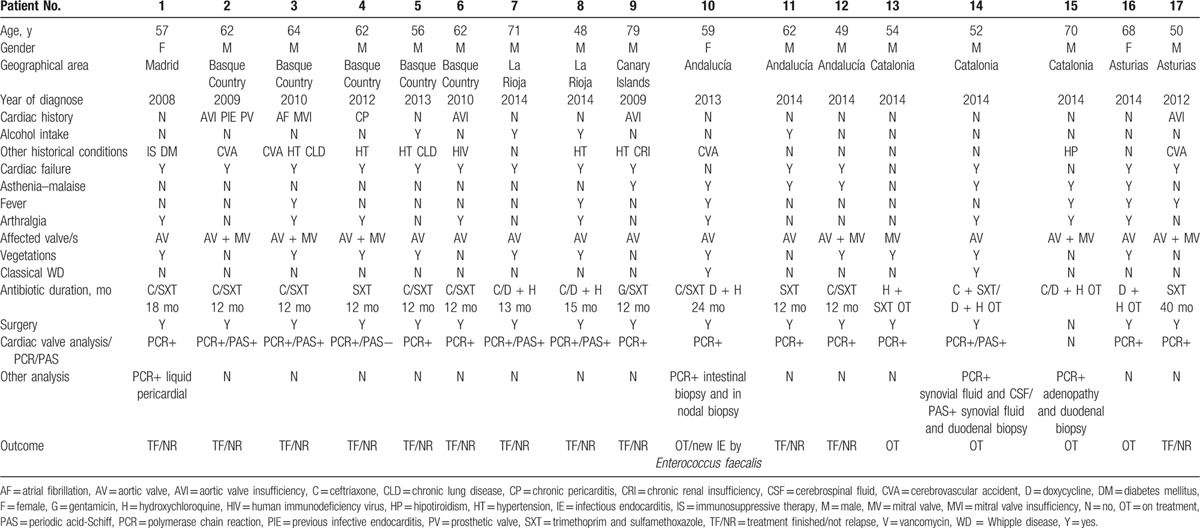
Main epidemiological, clinical, and outcome characteristics of the 17 patients with *T whipplei* endocarditis.

The mean age of *T. whipplei* endocarditis was 60.3 years (48–79 years) and most of cases were men, 14 (82.4%). Twelve patients (70.6%) were from the North of Spain, 1 from the Center (5.9%), 3 from the South (17.6%), and 1 (5.9%) from the Canary Islands.

### 3.2 Clinical features

According to the medical history, 6 patients had associated pathological cardiac conditions (35.3%): 4 had aortic insufficiency, 1 atrial fibrillation with mitral insufficiency, and 1 chronic pericarditis. One patient suffered a previous IE and carried a prosthetic valve. Of 17 patients, 5 had hypertension (29.4%), 4 patients (23.5%) suffered a cerebrovascular disease, and 2 patients (11.7%) chronic lung disease. Immunosuppressive therapy had been given only to 1 of the 17 patients (5.9%). One patient was HIV-positive (no AIDS) (5.9%). One had diabetes mellitus (5.9%) and 1 suffered of chronic renal insufficiency. Another 1 presented hypothyroidism and had been operated for knee osteoarthritis. Alcohol intake (>60 g/d) was referred by the 23.5% of the patients.

Cardiac failure was the main presenting form of *T. whipplei* endocarditis described in our patients. It was present in 13 of the 17 patients (76.5%). Another patient developed cardiac failure during the course of the illness. Chronic arthralgia was related in 9 of 17 patients (53%). Asthenia and malaise lasting more than 6 months were reported in 7 patients (41.2%). Fever was only recorded in 6 patients (35.3%). Only 2 patients presented classical WD (11.8%). One of them was diagnosed during the endocarditis process and the other, 1 month before.

Echocardiography was performed for all 17 patients: transthoracic echocardiography for 14 patients (82.4%) and transesophageal echocardiography for the same number. Echocardiography showed vegetations in 11 patients (64.7%). The valve involved was the aortic in 16 cases (94.1%), although 6 of them (37.5%) also presented mitral valve involvement. In 1 of the patients, the unique valve involved was the mitral. Native valve was affected in 16 patients (94.1%) while prosthetic valve was affected only in 1 case (5.9%). The size of the vegetations was among 5 and 33 mm but embolic phenomena were rare: only 1 patient (5.9%) developed a peripherical embolism (limb) in the course of the illness.

The main laboratory recording abnormalities at the time of the diagnosis were anemia which was detected in 88.2% of the patients (hemoglobin level average of 11 g/dL with a range between 9.7 and 12.4 g/dL) and an increasing of C-reactive protein with an average level of 51.8 mg/L (range: 2.3–136.7 mg/L). The glomerular filtration rate average was 66 mm/h (range: 26–98).

Surgery was performed in 16 patients (94.1%) and gave the definitive diagnosis of IE in 16/17 patients (94.1%) since none of the patients met the criteria for IE according to the Duke's university. The reasons of surgery were heart failure in 9 patients (56.3%) and severe regurgitation in 7 (43.8%). The other patient was diagnosed because of valve cardiac involvement in the context of classical WD.

### 3.3 Microbiological diagnosis

Culture of the valves was negative in all the cases. PAS staining was performed in valves from 6 patients (35.3%) with a positive result in 5 of them (83.3%). PCR against *T. whipplei* were positive in all studied valves with at least 2 different targets. An rRNA 16S PCR was used for first instance in all laboratories. Then, different targets were employed for confirmation (Table 2). PCRs were also positive in the intestinal biopsy in the 2 patients with classical WD and in the pericardial fluid of the patient with chronic pericarditis. In 1 of the patients affected by classical WD, a positive PCR in an adenopathy was also obtained. The patient in which cardiac valve surgery was not performed had a positive PCR in samples from small bowel, cerebrospinal fluid (CSF), and synovial fluid.

**Table 2:**
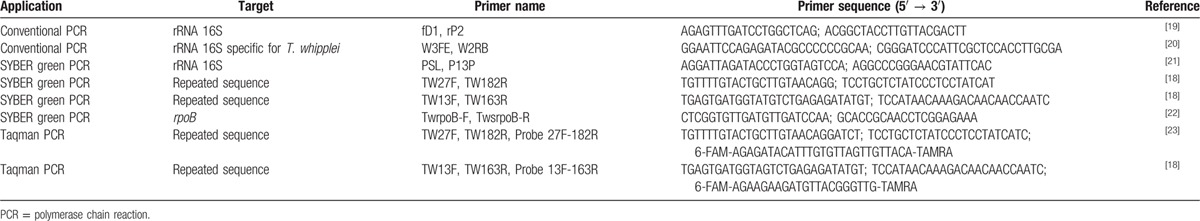
Primers used in this study.

### 3.4 Treatment and outcome

Eleven patients (64.7%) started treatment with ceftriaxone (2 g/IV at least for 2 weeks as initial therapy followed of different antimicrobials). Twelve patients (70.6%) received trimethoprim-sulfamethoxazole (SXT) 160/800 mg “bis in die” (BID) at least during 1 year (3 only SXT without another antimicrobial; 1 used gentamicin during the 1st week and other hydroxychloroquine). In 5 patients (29.4%), the elected antimicrobial drug was doxycycline 100 mg BID plus hydroxychloroquine 600 mg/d (1 of them first started with SXT and continued with doxycycline plus hydroxychloroquine). Details of the combinations are shown in Table 1. All regimens were administered during at least 1 year (average of treatment: 15.8 months).

The course during the treatment was satisfactory in all but 1 patients who suffered a new IE caused by an *Enterococcus faecalis*. None of the patients died during the IE process or during the follow-up after the end of treatment (Table 1). The follow-up after finishing the treatment has been from 2 to 65 months, with an average of 28.8 months.

## 4 Discussion

Although the suspicion and diagnosis of *T. whipplei* endocarditis can be difficult, more than 80 cases have been reported in the literature since 1997.^[12]^ Here we report 17 patients affected by *T. whipplei* endocarditis from a prospective cohort in Spain. This series joined to the French and German ones is the largest series of patients with *T. whipplei* endocarditis.^[13,14]^ Diagnosis of *T. whipplei* endocarditis remains a challenge due to this endocarditis does not exhibit the typical sings and blood cultures used to be negative. According to BCNE series, the rate of *T. whipplei* as causative agent of this condition is around the 0.6% to 2.6% of all the studied cases.^[4,24]^ These data are consistent with those presented in this article. Nevertheless, the prevalence of *T. whipplei* endocarditis could be underestimated due to the difficulties that involve the identification of *T. whipplei.* We do not know if the data shown in this article show the true incidence of *T. whipplei* endocarditis in our country, as it could happen in other countries, but we know that is a good approximation since the molecular study of removed valves is the rule in all hospitals of the GAMES cohort when IE is suspected. As in other studies, males (82.4%) are more frequently affected than females. The age of presentation (60.3 years) is also in the range of other forms of IE^[1]^ although less than in the registry of the total IE of the GAMES series (69 years).^[1]^ In the data shown in this work, an aggregation of cases can be observed in the North of Spain. A high prevalence of asymptomatic colonization that has been demonstrated in this area^[25]^ could explain this fact, but we do not know what can be the prevalence in other regions and a potential bias may occurs. However, such irregular distribution has been described in other countries where genetic host factors have been involved.^[14]^

As other authors have published, modified Duke criteria are ineffective for diagnosis before heart valve analysis.^[14]^ In our series, all but 1 patients were diagnosed after the affected valve was removed. Cardiac failure used to be the main presenting clinical debut form in *T. whipplei* endocarditis.^[12–14]^ Signs and symptoms in this entity are not the typical ones, and therefore, fever was present only in 35% of patients and embolic events in 5.9%. The presence of arthralgia as a prominent symptom varies depending on the series, showing values from 31% to 75% of the described cases.^[12–14]^ In our series, it has been 47% although in many cases we have looked for it when the diagnosis was made. This symptom is, sometimes, weak and only detected after an exhaustive clinical questioning. So, in patients with subacute endocarditis with negative blood cultures and low-grade fever (or not fever), if arthralgias are present, *T. whipplei* as causative agent should be suspected.^[14,26]^

Ecocardiography features are 1 of the most valuable tools for suspecting IE. In our series, echocardiography allowed the diagnosis of IE in 70.6%: visualization of vegetations in 11 and indirect signs in 1. In a previous report, we analyzed all the published cases in which these data were recorded and vegetations were seen in 64.3% of cases.^[12]^ In the French series,^[14]^ echocardiography showed vegetations in 78.6% of the patients, but these data are not recorded in the German one.^[13]^ In the reviewed literature, only 4 (4.7%) of the cases reported a classic WD concomitant with endocarditis^[12]^ and we have noticed it for 2 (11.8%) of cases. One of them was diagnosed during the IE process. In any case, to perform an echocardiogram should be made to these patients since endocarditis in the context of classic WD is more frequent than in other diseases and than in general population.

The histological study by PAS staining is considered a good tool for demonstrating WD. In our series, this technique was made in 6 patients and 5 of them demonstrate the PAS positive inclusions (sensitivity of 83.3%). Data of literature recorded PAS staining in 48 patients with similar results.^[12]^

Diagnosis of *T. whipplei* endocarditis in our series has been carried out with molecular tools on heart valve tissue. Different targets have been used for molecular analyses. PCR based on the 16S rRNA amplification and subsequent sequencing has been widely used and has been the first-line screening in our series. However, some authors alert that this broad-spectrum PCR could have a limited sensitivity (value sensitivity 60%, specificity 100%),^[27]^ while specific qPCR for *T. whipplei* have showed higher sensitivities.^[18,28]^ So, if 16S rRNA PCR has been negative, specific targets should be used in highly suspected cases of *T. whipplei*. At least 2 of the PCRs must be positive and their sequences have to show higher identity with the bacterium studied.

Current management of *T. whipplei* endocarditis is based from the experience acquired in the treatment of classical WD and in Q fever endocarditis.^[29,30]^ Most treatments used in *T. whipplei* endocarditis include 2 weeks of parenteral high dose of meropenem, penicillin G or ceftriaxone followed by an oral treatment strategy of 12 months with SXT (160/800 mg BID) or, at least, 18 months of doxycycline (100 mg BID) plus hydroxychloroquine (600 mg/d).^[10,29,30]^ Treatment of 2 weeks with ceftriaxone followed by 1 year with SXT has been the most recommended line,^[31]^ although in the recently published European guidelines^[6]^ for the management of infective endocarditis recommends doxycycline (100 mg BID) plus hydroxychloroquine (200–600 mg/24 h) orally for ≥18 months. This fact could be in relationship with the resistance observed in vitro of *T. whipplei* to trimethoprim^[32]^ and the reported case of a patient with clinically acquired resistance to SXT.^[33]^ In our series, most of patients have been treated with ceftriaxone for 2 weeks followed by SXT or with doxycycline plus hydroxychloroquine with good outcomes. Other combinations have been also employed with satisfactory results and none of our patients died during the IE process and neither had a relapse in the follow up.

After the end of treatment, some authors^[14]^ recommend the checking for the presence of *T. whipplei* in blood, saliva, and fecal samples every 6 months for 2 years and every year for the entire life of the patient. If colonization is detected, they recommend treating again, but there is not still evidence for this procedure.

In summary, *T. whipplei* IE is an infrequent condition that could be diagnosed with specific procedures (mostly molecular tests and thereafter PAS coloration) when culture negative IE undergo cardiac surgery. An early and appropriate diagnosis is required since this condition has a very good course and prognosis when the appropriate treatment is started. In our series, all patients who have finished the treatment have had good outcome and tolerance to the antimicrobial regimen used. Furthermore, we believe that in patients with unexplained valve destruction which requires cardiac surgery, an exhaustive clinical investigation must be performed and removed valves should be studied by molecular tools for to rule out an underlying IE.
